# Carnosine: can understanding its actions on energy metabolism and protein homeostasis inform its therapeutic potential?

**DOI:** 10.1186/1752-153X-7-38

**Published:** 2013-02-25

**Authors:** Alan R Hipkiss, Stephanie P Cartwright, Clare Bromley, Stephane R Gross, Roslyn M Bill

**Affiliations:** 1 Aston Research Centre for Healthy Ageing, School of Life and Health Sciences, Aston University, Birmingham B4 7ET, UK

**Keywords:** Carnosine, Energy metabolism, Reactive oxygen species (ROS), Methylglyoxal, Proteolysis, Alzheimer’s disease, Parkinson’s disease, Diabetes, Cancer, Yeast

## Abstract

The dipeptide carnosine (β-alanyl-L-histidine) has contrasting but beneficial effects on cellular activity. It delays cellular senescence and rejuvenates cultured senescent mammalian cells. However, it also inhibits the growth of cultured tumour cells. Based on studies in several organisms, we speculate that carnosine exerts these apparently opposing actions by affecting energy metabolism and/or protein homeostasis (proteostasis). Specific effects on energy metabolism include the dipeptide’s influence on cellular ATP concentrations. Carnosine’s ability to reduce the formation of altered proteins (typically adducts of methylglyoxal) and enhance proteolysis of aberrant polypeptides is indicative of its influence on proteostasis. Furthermore these dual actions might provide a rationale for the use of carnosine in the treatment or prevention of diverse age-related conditions where energy metabolism or proteostasis are compromised. These include cancer, Alzheimer's disease, Parkinson's disease and the complications of type-2 diabetes (nephropathy, cataracts, stroke and pain), which might all benefit from knowledge of carnosine’s mode of action on human cells.

## Review

### Carnosine and cellular ageing

**Figure 1:**
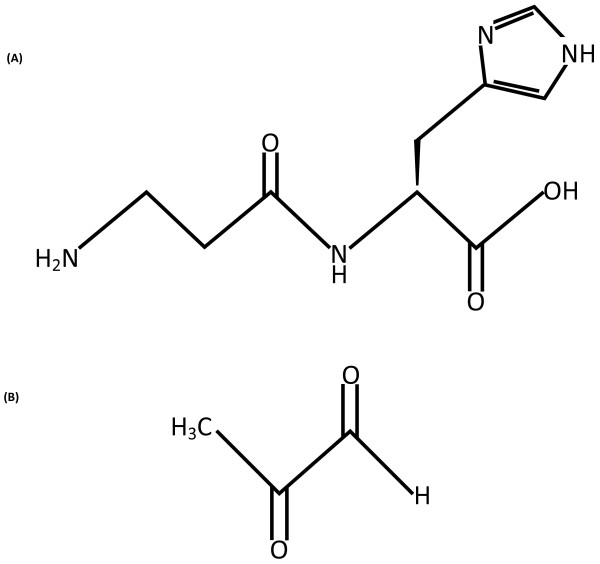
(A) Structure of L-carnosine, the dipeptide β-alanyl-L-histidine; (B) structure of methylgloxal (2-oxopropanal).

In 1994, McFarland and Holliday demonstrated that when the naturally-occurring dipeptide, carnosine (Figure 1), was added to cultures of primary human fibroblast cells, chronological lifespan was increased; the onset of senescence was effectively delayed in these cells [1]. Carnosine addition was also observed to rejuvenate already senescent cells, giving them a more juvenile appearance [1]. Paradoxically, a subsequent study revealed that carnosine selectively inhibited the growth of cancer cells, at least in culture [2]. Since explanatory mechanisms for these seemingly opposing effects are still unknown, carnosine has been called enigmatic [3].

Carnosine (β-alanyl-L-histidine) was discovered over 100 years ago (see [4] for a historic account). It occurs naturally in the brain, kidney and skeletal muscle of fish, birds and mammals at concentrations sometimes as high as 100 mmol kg^-1^ dry muscle mass [5]. Functionally carnosine appears to be pluripotent as there is evidence that it can scavenge reactive oxygen species (ROS) [6] and reactive nitrogen species (RNS) [7], can form adducts with deleterious aldehydes and ketones [8-11] and can act as a metal ion chelator [12] and hydrogen ion buffer [13]. Carnosine has also been demonstrated to affect gene expression [14], protein phosphorylation [15] and, possibly, mRNA translation initiation through the regulation of the eukaryotic initiation factor 4E protein (eIF4E) [16]. Despite this range of properties, the actual physiological function of carnosine remains unknown.

The addition of carnosine to cells has been shown to result in three outcomes that are characteristic of long-lived model systems [17]. These are decreased glycolysis, increased mitochondrial activity and suppression of proteotoxicity [17]. While these observations may hint at which of carnosine’s diverse properties are responsible for increasing chronological lifespan, any mechanistic rationale must also account for carnosine’s selective toxicity towards tumour cells. In this review, we discuss mechanisms that could accommodate the uniquely disparate effects of carnosine on cellular activity.

### Carnosine and changes in energy metabolism

#### Tumour cells, carnosine and glycolysis

**Figure 2:**
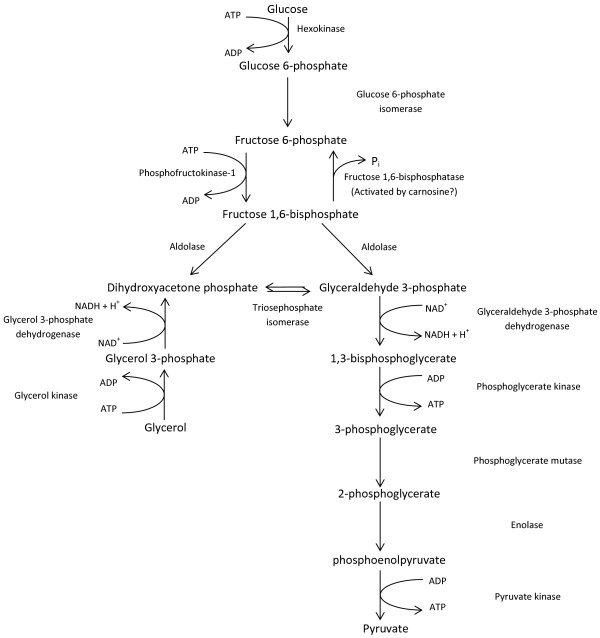
**An overview of glycolysis by which the conversion of glucose to pyruvate is coupled to the production of ATP for energy and NADH for biosynthesis.** The entry of glycerol into the glycolytic pathway is also shown. The scheme indicates the hypothetical action of carnosine in the activation of fructose 1,6-bisphosphatase to create a futile, ATP-consuming, cycle which also inhibits glycolytic ATP generation.

The metabolism of tumour cells is characteristically shifted towards cytosolic glycolysis [18,19], as first reported by Otto Warburg [20]. However, respiratory activity is not necessarily compromised [21,22] and has recently been proposed to be central to cancer progression [23]. Consensus has yet to be reached on the reasons for these complex metabolic switches, but the high energy and macromolecular precursor demands of rapidly growing tumours may provide an explanation [24]. We speculate that carnosine’s effects on tumour cells might be explained, in part, by its action on glycolysis (Figure 2). For example, whilst investigating the influence of carnosine on cultured brain tumour cells, Gaunitz and co-workers discovered that its addition inhibited cell growth due to the large decline in glycolytically-synthesized ATP [25,26]. Our own study in yeast (which can be used to model cancer cells [24]) led to the conclusion that carnosine may affect glycolysis [27]; addition of carnosine to yeast growing on glucose as sole carbon source, where the majority of ATP is generated from glycolysis, caused up to 20% cell death and a decreased overall growth rate. In contrast, cells growing aerobically on glycerol as sole carbon source were not inhibited by the addition of carnosine and showed an increased growth rate. Because glycerol is normally metabolized via dihydroxyacetone phosphate (DHAP) and glyceraldehyde 3-phosphate (G3P), these observations could support an interpretation that carnosine inhibits glycolysis prior to the formation of these triose phosphates from their glycolytic precursor, fructose 1,6-bisphosphate (Figure 2).

Unfortunately there is no evidence in the literature to demonstrate a direct effect on glycolysis by carnosine. However, in 1980, *in vitro* experiments on rabbit muscle demonstrated that both carnosine and histidine stimulate the activity of fructose 1,6-bisphosphatase (FBPase), which converts fructose 1,6-bisphosphate to fructose 6-phosphate [28] (Figure 2). The mechanism of this stimulation is unknown but, in the case of carnosine, could potentially be due to its ability to chelate the metal ions (such as Zn^2+^ and Mg^2+^[12]), that regulate glycolytic enzymes [29]. For example, if carnosine addition were to activate FBPase *in vivo* by chelating Zn^2+^[28], this would create a futile, ATP-consuming cycle since the ATP-utilizing enzyme phosphofructokinase converts fructose 6-phosphate into fructose 1,6-bisphosphate (Figure 2). This cycle would decrease ATP levels and ATP synthesis as well as decreasing the supply of carbon skeletons for amino acid synthesis. While this hypothesis is inconsistent with the fact that addition of histidine does not result in the death of glucose-grown yeast cells [27], it remains conceivable that carnosine’s metal-chelating properties influence the function of one or more glycolytic enzymes.

#### Carnosine and the metabolism of ageing cells

The metabolic shifts that occur as organisms grow, mature and finally age are complex and incompletely understood. When rapid growth ceases, in the transition to adulthood, the preferred pathway for ATP generation changes from glycolysis to oxidative phosphorylation [17]. However, one hallmark of cellular ageing is increased mitochondrial dysfunction; this frequently leads to cells reverting to glycolysis for ATP generation [30]. Consequently, it is likely that a subtle balance in the regulation of glycolysis and oxidative phosphorylation is critical throughout the lifespan [31].

Literature reports indicate that post-mitotic, adult (and therefore typically less glycolytic) cells have higher carnosine concentrations than actively-dividing cells, although the reasons for this tendency are unknown. For example, during murine brain development, carnosine synthesis is only associated with the final stages of glial cell maturation [32]. Carnosine is also present only in post-mitotic retinal neurones [33] when energy metabolism switches from glycolysis to oxidative phosphorylation [31]. In children, muscle carnosine levels are initially quite low (30–40 mg%) at 5 years of age but, as they grow, gradually increase to 120–140 mg% at 14 years of age [34,35] before declining and reaching a plateau in adulthood. Together these observations might suggest that carnosine is beneficial to adult cells (which employ oxidative phosphorylation for ATP generation), whereas in growing cells (which employ glycolysis to provide metabolic precursors and ATP), carnosine could even be detrimental. However, contrary to this suggestion, carnosine concentrations are higher in fast-twitch, glycolytic muscle than in slow-twitch, aerobic muscle [36]; this observation argues against the proposition that carnosine is more beneficial to aerobic cells than those that employ glycolysis to synthesize ATP. While any correlation between carnosine concentrations and metabolic state is unlikely to be clear cut, it has been suggested that high carnosine levels in adult (but not senescent) glycolytic tissue are required to maintain pH by buffering the high amounts of protons produced as a consequence of glycolytic activity (e.g. through lactic acid formation) and to combat the potentially deleterious by-products of glycolysis such as methylglyoxal (MG; Figure 1) [9].

It has also been noted that addition of carnosine to cultured rat fibroblasts strongly stimulates synthesis of the cytoskeletal protein, vimentin [14]; vimentin is closely, but not exclusively, involved with mitochondrial movement and localization [37]. Carnosine has also been observed to have a beneficial but unspecified organisational effect towards mitochondria [38]. One possibility is that the stimulation of vimentin synthesis by carnosine may in turn assist mitochondrial synthesis and intracellular targeting in ageing cells. These observations might support an interpretation that carnosine is associated with the metabolic rewiring that occurs when rapid growth declines and finally ceases, a change that is often accompanied by decreased glycolysis and increased mitochondrial activity. If carnosine were to positively influence mitochondrial development or activity, and also provide protection against deleterious glycolytic by-products (e.g. MG, especially following the reversion to glycolysis resulting from age-related mitochondrial damage in senescent tissues), this might help to explain the dipeptide's rejuvenating effects on senescent cultured human fibroblasts [1]; currently, this hypothesis remains to be tested.

### Carnosine and age-related changes in proteostasis

**Figure 3:**
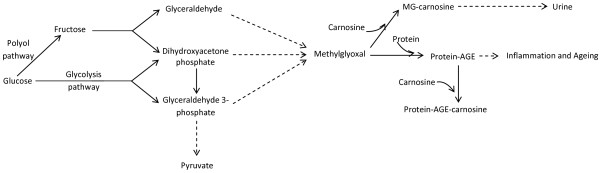
**Metabolic sources of methylglyoxal (MG) and the possible role of carnosine in scavenging MG and suppressing the formation of protein-AGEs.** Protein-AGEs cause inflammation and ageing. MG-carnosine is excreted in urine.

Increased proteolytic activities (autophagic and proteasomal [39,40]) and the up-regulation of one or more heat shock and/or chaperone proteins are associated with lifespan extension in yeast [41], birds and mammals [42]; they help to maintain proteostasis by degrading altered proteins. Conversely, the accumulation of altered proteins (proteostatic dysfunction) is a major hallmark of ageing [43]. MG (Figure 1) is a well-characterized α-ketoaldehyde whose toxic effects on cells and tissues closely mimic those of the ageing process. When serum glucose levels are raised, MG is increased [44] and is increasingly regarded as a major source of age-related protein damage and proteoxicity [45,46] as it can form adducts, known as advanced glycation end-products (AGEs; Figure 3), with lysine, histidine, arginine and cysteine residues of target proteins. Increased formation of MG may also arise via the polyol pathway [47], which also generates glyceraldehyde and DHAP. If not immediately metabolized to 1,3-bisphosphoglycerate, these trioses spontaneously decompose into MG (Figure 3). Carnosine’s ability to scavenge reactive species such as MG (and others such as malondialdehyde, a lipid peroxidation product) is well documented [8,9,17,48] and might explain the dipeptide’s ability to delay cellular senescence.

There is evidence that carnosine, either as the free dipeptide or when complexed with zinc ions (so-called polaprezinc), can induce expression of the heat shock proteins, Hsp 27 [49] and Hsp70/72 [50]. Other studies have also demonstrated that carnosine can stimulate a cytosolic protease [51] or indeed proteolysis of long-lived proteins in senescent cultured human fibroblasts [52]. Although these findings (some preliminary) seem to suggest that carnosine might help to maintain proteostasis, further experimentation is required to confirm this hypothesis.

**Figure 4:**
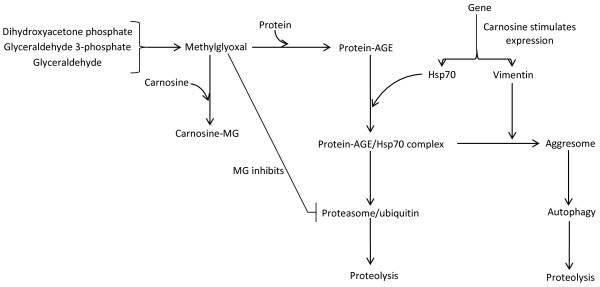
**The possible effects of carnosine on the formation and catabolism of abnormal proteins.** MG, methyglyoxal; AGE, advanced glycation end-product; Hsp70, heat shock protein 70 (shown as an example).

The stimulation of vimentin synthesis in cultured rat fibroblasts by carnosine [14] may be relevant in this context as well. Vimentin has been suggested to participate in the formation of aggresomes into which protein aggregates are sequestered, especially when proteasomal activity is inhibited [53]; it has been proposed that vimentin forms a cage surrounding the target protein (which is frequently ubiquitinated) [54]. The enzyme, oxidized protein hydrolase (OPH), is co-expressed with vimentin [55] raising the possiblity that OPH and vimentin co-operate to form aggresomes, which, together with proteasomes, facilitate the disposal of oxidized proteins [55] and thereby help to maintain proteostasis (Figure 4).

Recent studies have confirmed the view that maintenance of proteolytic function is important for regulating energy metabolism: 6-phosphofructo-2-kinase/fructose 2,6-bisphosphatase (Pfkfb3), which generates fructose 2,6-bisphosphate, is subject to continuous proteasome-mediated degradation following its ubiquitination [56,57]. However, if degradation of Pfkfb3 is inhibited, glycolysis is stimulated and oxidative stress results [56]; in neurones this provokes cell death [57]. Possible explanations for these findings are a reduced pentose phosphate pathway metabolism and/or increased MG formation following increased triose phosphate formation. Collectively these observations illustrate how proteostatic dysfunction can directly impact energy metabolism and *vice versa*[58]; excess MG formation may compromise the function of the ubiquitin/proteasome system [44], illustrating the delicate interdependence between energy metabolism and proteostasis.

### The therapeutic potential of carnosine

As carnosine may possess activities that suppress age-related dysfunction in either energy metabolism or proteostasis [17], it has been proposed as a potential therapeutic agent [see [59] for recent review]. Indeed carnosine may resemble other naturally-occurring agents, such as resveratrol, that possess similar pluripotency [60] and therefore have the potential to be used as “smart drugs” that simultaneously act on multiple targets [61]. Carnosine is also well-documented to have antioxidant properties (see [4] for more information): it can scavenge reactive oxygen species such as hydroxyl radicals, superoxide and singlet oxygen [62]. Moreover, carnosine can chelate the heavy metals that cause cellular damage [63]. These properties protect macromolecules, such as lipids, proteins and DNA, from the damage which results in ageing and age-related disease [64].

While there are no current reports of clinical trials concerning carnosine’s affects towards clinically-defined age-related dysfunction, it was recently reported that carnosine improved cognition in schizophrenics [65]. In senescence-accelerated mice, a diet supplemented with carnosine decreased the signs of ageing and increase mean lifespan by 20% [66].

#### Cancer

The ability of carnosine to suppress the growth of tumour cells has recently been reviewed [67], one possible mechanism being its inhibition of energy metabolism. Consistent with this is the finding that carnosine inhibits glycolytic ATP generation [25], although the mechanistic details underpinning this observation remain to be determined. Possibilities include the stimulation of FBPase activity, described above, effects on another glycolytic enzyme or its effects on the intracellular signalling cascades that regulate translational processes. For example, a reduction in phosphorylation of the translation initiation factor eIF4E [16] might alter the rate of mRNA initiation and consequently of protein synthesis [68]. Although a full picture is not currently available of the different intracellular mechanisms at play following carnosine treatment, initial evidence suggests that both mitogen-activated (MAP) kinase and mammalian target of rapamycin complex (mTOR) may be involved [16]. Our own work in *Saccharomyces cerevisiae* supports this idea since we have demonstrated that deletion of *TOR1* confers resistance to carnosine treatment [27]. Further work is needed to decipher how eIF4E activities might be regulated in response to carnosine. The eIF4E binding protein (eIF4E-BP) is of particular interest as it is known to be regulated by mTOR and is affected by the well-known anti-tumour and anti-ageing agents, rapamycin [69] and resveratrol [70].

AGEs, which are formed at an accelerated rate during normal ageing and in diabetics, have been shown to have a role in the development and progression of cancers; it is believed that through interaction with their receptor (RAGE) they stimulate pro-inflammatory gene activation and hence oxidative stress [71,72]. We have shown that carnosine inhibits the formation of AGEs: it can protect against MG modification [9] and significantly reduces the formation of protein-crosslinking and oxidative modification [59]. The ability of carnosine to prevent AGE formation has also been demonstrated by others both *in vitro*[73-75] and *in vivo*[48]; the plethora of signalling cascades activated include NF-κB, MAPKs, PI3K/Akt and the Rho GTPases. Direct evidence for a role for carnosine and AGE/RAGE in tumorigenesis is currently missing, but may provide new avenues of research to inhibit tumour growth.

#### Alzheimer’s disease

There is extensive evidence from animal studies showing that carnosine is a neuroprotective agent [76]. Further evidence suggesting that carnosine might be used to control Alzheimer’s disease (AD) [77,78] includes its ability to suppress the toxic effects of amyloid beta towards cultured cells [79,80] and to inhibit sugar-dependent protein aggregation [81]. Importantly carnosine was found to suppress the accumulation of amyloid in transgenic mice [82]; the mechanisms responsible are uncertain but could involve zinc ion modulation, up-regulation of heat shock protein expression, and/or enhanced proteolysis of the aberrant polypeptide. As there is a strong link between type-2 diabetes and AD [60], the ability of carnosine to suppress glycation-related phenomena should also be explored in relation to AD [59].

Carnosine has been shown to suppress mitochondrial dysfunction in a transgenic mouse model of Alzheimer’s disease [82] and is also an activator of carbonic anhydrase (CA), which is decreased in AZ patients [83]. The activity of some CA isozymes has been reported to decline in certain parts of the human brain with age [84] and silencing of the CA gene, *cah-3*, in the nematode *Caenorhabditis elegans* is reported to reduce lifespan [85].

#### Parkinson’s disease

Preliminary studies have demonstrated beneficial effects of carnosine supplementation in PD patients [86,87]. Very recently it was found that in the brains of PD patients, the substantia nigra (the area subject to degeneration in PD), contains up to 3 times more non-specific cytosolic carnosinase, CNDP2 [88], compared to controls. If carnosine is normally protective in the substantia nigra, raised cellular carnosinase activity would lower that protection. This interpretation is supported by the finding that high glycemic index diets in mice raise MG-damaged protein levels in the substantia nigra [44]. That the substantia nigra is particularly susceptible to degeneration may derive from the fact that it synthesizes dopamine; it was recently shown that MG can spontaneously react with dopamine to produce 1-acetyl-6,7-dihydroxy-1,2,3,4-tetrahydroisoquinoline (ADTIQ), which is found in brains affected by PD [89]. Importantly, carnosine has been shown to inhibit ADTIQ toxicity [90]. It is also possible that carnosine, by scavenging MG, could additionally inhibit ADTIQ formation. Overall, these findings suggest that carnosine could possess therapeutic potential towards PD [91].

#### Diabetes-related diseases

In type-2 diabetes patients, cataractogenesis [92], diabetic kidney disease [93] and atherosclerosis are common consequences [94] of MG-induced glycation of proteins, as well as other cellular dysfunction. It has also been recognised for some time that there is a relationship, possibly causal, between type-2 diabetes and AD [60]. A similar association may exist for type-2 diabetes and other age-related diseases such as PD [95-97]; again MG could be a major contributing or even causal factor [98].

As carnosine has been shown to exert protective activity against protein modification mediated by MG and other reactive carbonyls, the dipeptide has been explored for its therapeutic potential towards complications associated with type-2 diabetes [99]. Two recent studies have shown that diabetes-related peripheral algesia (pain) is mediated by the generation of MG in neural tissue [100,101]. Carnosine has been shown to possess anti-nociceptive activity (pain suppression) in mice [102,103], which could be due to the dipeptide’s ability to react with MG. Hence it is possible that carnosine could be one of several therapeutic options in alleviating diabetes-related pain.

### Carnosine administration: overcoming the carnosinase problem

It is often thought that the presence of serum carnosinase in humans is an impediment to the therapeutic use of carnosine [59]; indeed reduced levels of carnosinase in serum have been suggested to reduce diabetic complications [104]. However, a number of strategies could overcome this perceived obstacle. These include using modified forms of L-carnosine resistant to carnosinase attack (e.g. N-acetyl-carnosine); using an intra-nasal delivery route to combat neurodegeneration and brain tumours; and using N-acetyl-carnosine-containing eye drops, which could be employed for treating cataracts.

## Conclusions

Carnosine appears to have metabolism-dependent effects on cells and may inhibit ATP production during glycolysis. Carnosine also appears to facilitate the selective elimination of aberrant polypeptides and may stimulate the synthesis of stress proteins, thereby helping to maintain the proteome. By influencing two fundamental biochemical characteristics of the aged phenotype, energy metabolism and proteostasis, this intriguing dipeptide has the potential to ameliorate a range of age-related conditions.

## Abbreviations

AD: Alzheimer’s disease; ADTIQ: 1-acetyl-6,7-dihydroxy-1,2,3,4-tetrahydroisoquinoline; CA: Carbonic anhydrase; DHAP: Dihydroxyacetone phosphate; eIF4E: Eukaryotic initiation factor 4E protein; FBPase: Fructose 1,6-bisphosphatase; G3P: Glyceraldehyde 3-phosphate; MAP: Mitogen-activated kinase; MG: Methylglyoxal; mTOR: Mammalian target of rapamycin complex; OPH: Oxidized protein hydrolase; PD: Parkinson’s disease; Pfkfb3: 6-phosphofructo-2-kinase/fructose 2,6-bisphosphatase.

## Competing interests

The authors declare that they have no competing interests.

## Authors’ contributions

All authors contributed to drafting the manuscript. All authors read and approved the final manuscript.

## Authors’ information

ARH is a visiting researcher at Aston Research Centre for Healthy Ageing (ARCHA); SPC is a PhD student supported by a BBSRC Targeted Priority Studentship in Ageing in RMB’s laboratory; CB is an undergraduate student studying Biomedical Sciences at Aston University, on a placement year in RMB’s laboratory; SRG is a Lecturer in Cell Biology in the School of Life and Health Sciences at Aston University; RMB is Professor of Biotechnology in the School of Life and Health Sciences at Aston University and sits on ARCHA’s Internal Advisory Board.
